# Enhanced Therapeutic Effects of ^177^Lu-DOTA-M5A in Combination with Heat Shock Protein 90 Inhibitor Onalespib in Colorectal Cancer Xenografts

**DOI:** 10.3390/cancers15174239

**Published:** 2023-08-24

**Authors:** Tabassom Mohajershojai, Douglas Spangler, Saloni Chopra, Fredrik Y. Frejd, Paul J. Yazaki, Marika Nestor

**Affiliations:** 1 Department of Immunology, Genetics and Pathology, Uppsala University, 751 85 Uppsala, Sweden; tabassom.mohajer@igp.uu.se (T.M.); saloni.chopra@igp.uu.se (S.C.); fredrik.frejd@igp.uu.se (F.Y.F.); 2 Department of Public Health and Caring Sciences, Uppsala University, 751 22 Uppsala, Sweden; douglas.spangler@pubcare.uu.se; 3 Department of Immunology & Theranostics, Beckman Research Institute of the City of Hope, Duarte, CA 91010, USA; pyazaki@coh.org

**Keywords:** radioimmunotherapy, combination therapy, carcinoembryonic antigen, ^177^Lu-DOTA-M5A, HSP90 inhibitor onalespib

## Abstract

**Simple Summary:**

Cancer treatment is hampered by the limitations of individual therapy modalities and the intricate nature of the disease. The administration of maximal monotherapy doses often leads to undesirable side effects and/or therapy resistance. As a result, there is a growing recognition of the importance of investigating combination therapy to effectively address these obstacles. In the present *in vivo* study, the therapeutic effects of combination therapy with the heat shock protein 90 inhibitor onalespib, a potential radiosensitizer, and ^177^Lu-DOTA-M5A for colorectal cancer (CRC) treatment were explored for the first time. The results demonstrated that the combination treatment was so effective that retained or even superior therapeutic effects could be achieved with only half the dose of administered ^177^Lu-DOTA-M5A, showing enhanced tumor growth suppression and increased apoptosis. Consequently, the combination therapy involving ^177^Lu-DOTA-M5A and onalespib constitutes a promising approach for treating metastatic CRCs. By enhancing therapeutic effects, minimizing therapy resistance, and reducing side effects, this approach has the potential to expand the patient population that can benefit from targeted treatment.

**Abstract:**

Carcinoembryonic antigen (CEA) has emerged as an attractive target for theranostic applications in colorectal cancers (CRCs). In the present study, the humanized anti-CEA antibody hT84.66-M5A (M5A) was labeled with ^177^Lu for potential CRC therapy. Moreover, the novel combination of ^177^Lu-DOTA-M5A with the heat shock protein 90 inhibitor onalespib, suggested to mediate radiosensitizing properties, was assessed *in vivo* for the first time. M5A antibody uptake and therapeutic effects, alone or in combination with onalespib, were assessed in human CRC xenografts and visualized using SPECT/CT imaging. Although both ^177^Lu-DOTA-M5A and onalespib monotherapies effectively reduced tumor growth rates, the combination therapy demonstrated the most substantial impact, achieving a fourfold reduction in tumor growth compared to the control group. Median survival increased by 33% compared to ^177^Lu-DOTA-M5A alone, and tripled compared to control and onalespib groups. Importantly, combination therapy yielded comparable or superior effects to the double dose of ^177^Lu-DOTA-M5A monotherapy. ^177^Lu-DOTA-M5A increased apoptotic cell levels, indicating its potential to induce tumor cell death. These findings show promise for ^177^Lu-DOTA-M5A as a CRC therapeutic agent, and its combination with onalespib could significantly enhance treatment efficacy. Further *in vivo* studies are warranted to validate these findings fully and explore the treatment’s potential for clinical use.

## 1. Introduction

Colorectal cancer (CRC) is the third most prevalent cancer worldwide, with over 1.9 million cases diagnosed and 0.9 million deaths reported annually [1,2]. Carcinoembryonic antigen (CEA) is a cancer-associated biomarker and is highly expressed in colorectal tumors as well as other solid tumors such as gastric, pancreatic, breast, and lung cancer [3,4,5]. CEA undergoes post-translational modification known as glycosylphosphatidylinositol (GPI) anchoring, leading to the attachment of a phosphoglyceride moiety to its C-terminus. This specific molecular event renders CEA a membrane-bound protein with GPI-anchored localization. The biological, physiological, and pathological functions of CEA is not yet fully understood. There are studies suggesting a role in cell adhesion, where cells are more loosely associated with each other in normal tissues [6], and a potential role in innate immune defense of the colon and other tissues from microbial attack [7,8]. In the domain of pathological functions of CEA, there are studies suggesting a role in tumorogenesis function *in vitro* [9] and in transgenic mice [10,11]. CEA is a GPI membrane-bound protein that is minimally expressed on the apical surface of normal colorectal epithelial cells, but its expression is elevated on the basolateral surface of CRC cells that are accessible to the bloodstream [12,13,14,15,16]. Higher expression of CEA on cancer cells leads to higher levels of circulating CEA in blood, and the association between CEA serum level and prognosis of the disease has been widely studied [17]. Consequently, CEA has become a preferred marker for diagnosis and prognosis of CRC, and it is also a promising target for CRC research and therapy [12,18,19,20].

Radioimmunotherapy (RIT) using radiolabeled conjugates to target CEA is a promising approach to the treatment of cancers including CRCs. Radiolabeled antibodies such as T84.66 have shown positive outcomes *in vitro*, *in vivo*, and in clinical trials [21]. The chimeric cT84.66 has been studied extensively as a single agent and with chemotherapies [4,21,22,23,24], and a humanized version (M5A) was developed to minimize immunogenicity [25]. A phase I clinical trial with ^90^Y-labeled M5A in patients with advanced CEA-expressing tumors showed therapeutic effects, feasibility of therapy, and antibody tolerance [14].

^177^Lu is a promising therapeutic radionuclide for targeted therapy, and presents lower energy and a shorter emission range compared to ^90^Y [26]. Recently, treatment of 3D *in vitro* models with ^177^Lu-DOTA-M5A demonstrated promising therapeutic effects on colorectal cancer models [27]. The therapeutic effects of ^177^Lu-DOTA-M5A have, however, not been studied *in vivo*, and this study constitutes the first *in vivo* assessment of the therapeutic effects of ^177^Lu-DOTA-M5A.

A second target of interest in CRCs is heat shock proteins (HSPs), a family of molecular chaperones that play pivotal roles in the correct folding and function of client proteins [28]. In particular, HSP90 and its client proteins are involved in numerous cancer hallmarks, including proliferation, metastasis, and invasion [29,30]. The HSP90 client proteins comprise unstable signaling molecules such as kinases, transcription factors, and chromatin remodeling. This can eventually result in cancer cells gaining capabilities of self-sufficiency in growth signaling, insensitivity to anti-growth signaling, limitless replicative potential, and eventually invasion and metastasis [31,32]. High expression of HSP90 is associated with a poor prognosis in colorectal cancer patients [33] as well as other types of malignancies such as melanoma, leukemia, lung, and breast cancers [34,35,36,37]. By inhibiting HSP90, cancer progression can be attenuated, as many HSP90 client proteins and related pathways are required for cancer cell progression. Onalespib is a second-generation HSP90 inhibitor with better solubility and less hepatotoxicity than previous inhibitors such as 17-AAG and 17-DMAG. HSP90 inhibition has also been shown to delay DNA repair mechanisms and the repair of radiation-induced double strand DNA breaks (DSBs) [38,39].

The therapeutic effects of onalespib have been demonstrated in several preclinical and clinical studies. Combination therapies of onalespib and chemotherapy drugs/radiation in solid tumors have demonstrated greater therapeutic effects compared to monotherapies [40,41]. Onalespib, when combined with external beam radiation or ^177^Lu-DOTATATE, suggested radiosensitizing properties in colorectal cancer and neuroendocrine cancer models [38,42]. Moreover, an *in vitro* study using colorectal 3D spheroid models demonstrated synergistic therapeutic effects of onalespib combined with ^177^Lu-DOTA-M5A [27]. Thus, the radiosensitizing properties of onalespib in preclinical studies and particularly in colorectal cancers, as well as the pronounced effects of HSP90 inhibitors on various cellular pathways motivate the investigation of the combination of ^177^Lu-DOTA-M5A and onalespib in colorectal cancer.

The aim of the current study was to assess the *in vivo* effects of ^177^Lu-DOTA-M5A therapy and HSP90 inhibition in colorectal cancer xenografts and to further investigate the radiosensitizing potential of onalespib when combined with ^177^Lu-DOTA-M5A. The therapeutic effects and possible toxicities were assessed in mice bearing human colorectal cancer xenografts.

## 2. Materials and Methods

### 2.1. Cell Culture and Maintenance

The human colon carcinoma CEA-positive, onalespib-sensitive cell line HT55 was obtained from the European Collection of Authenticated Cell Culture (ECACC [43]), and the colon adenocarcinoma CEA-positive, onalespib-sensitive cell line SNU1544 was obtained from Korean Cell Line Bank (KCLB [44,45]). CEA expression level and onalespib sensitivity in both cell lines have been previously assessed [27,46]. The HT55 cells were cultured in Minimum Essential Medium (MEM) (Biowest, Riverside, MO, USA) supplemented with 20% (*v*/*v*) fetal bovine serum (FBS; Sigma Aldrich, St. Louis, MO, USA). The SNU1544 cell line was cultured in RPMI [(Biowest, MO, USA, containing 25 nM N-2-Hydroxyethylpiperazine-N’-2-Ethanesulfonic Acid (HEPES)] supplemented with 10% (*v*/*v*) heat-inactivated FBS (Sigma Aldrich, MO, USA). Heat inactivation was done at 56 °C for 30 min. All media were supplemented with antibiotics (100 IU penicillin and 100 µg/mL streptomycin, Biochrom GmbH, Berlin, Germany) and L-glutamine (Biochrom GmbH, Berlin, Germany, 2 mM). Monolayer cultures were grown in tissue culture flasks (VWR, Radnor, PA, USA) and incubated in an atmosphere containing 5% CO_2_ at 37 °C. After reaching 75–85% confluency, cell passaging was performed using Trypsin-EDTA (Biochrom GmbH, Germany). All cell lines were cultured less than 3 months after purchase.

### 2.2. M5A Monoclonal Antibody, Radiolabeling, and Onalespib

The anti-CEA hT84.66-M5A mAb is a humanized IgG_1_ [25]. To select and optimize the suitable xenograft model, the M5A mAb was labeled with ^125^I using 12 × 75 mm pre-coated glass iodogen tubes (50 μg of iodogen/tube) according to the manufacturer’s instruction (Thermofisher Scientific, Waltham, MA, USA). Briefly, the iodogen tube was washed with 1 mL of Phosphate Buffered Saline (PBS, St. Louis, MO, USA) followed by adding 100 μL of PBS. The ^125^I was activated by adding 5 MBq of ^125^I to PBS, mixing by pipetting and incubating for 6 min at room temperature (swirling every 30 s). The activated ^125^I was then transferred to a new tube along with 50 μg of M5A antibody. The mixture of M5A and ^125^I was stirred on a thermoshaker (TS-100C Smart, Biosan, Riga, Lativa) at 37 °C for 15 min (350 rpm), yielding the final specific activity of 100 kBq/μg. The extent of radiolabeling yield was assessed using instant thin layer chromatography (ITLC, Biodex Medical Systems, New York, NY, USA) and NaCl (0.9%) as a mobile phase, and the radiolabeling yield was quantified using a phosphorimager (BAS-1800II, Fujifilm, Tokyo, Japan). For the biodistribution experiment with ^125^I-M5A, injected activity was 250 kBq and unlabeled M5A was added to obtain 150 μg/mL.

The M5A mAb was conjugated with NHS-DOTA, purified as previously described [47], and labeled with ^177^Lu for biodistribution and therapy studies [27]. Briefly, DOTA-M5A mAb was buffer exchanged to sodium acetate (0.2 M, pH 5.5) using a spin column (Amicon Ultra-0.5 mL, 3 kDa NMWCO, Merck, Darmstadt, Germany). The mixture of DOTA-M5A (50 μg) dissolved in sodium acetate (0.2 M, pH 5.5) and ^177^Lu (15 MBq, in 0.04 M HCl, ITG GmbH) was stirred on the thermoshaker at 42 °C for 1 h (350 rpm), yielding the final specific activity of 300 kBq/μg. The extent of radiolabeling yield was evaluated using ITLC and citric acid (0.2 M, pH 5.5) as a mobile phase and the radiolabeling yield was quantified using a phosphorimager. The ^177^Lu-DOTA-M5A was purified using a spin column. A labeling yield above 96% or higher was used for animal experiments. ^177^Lu-DOTA-M5A was further diluted in Dulbecco’s Phosphate Buffered Saline (DPBS, Biowest, MO, USA) to 150 μg/mL and 100 μL was injected intravenously (IV) in the tail vein. For assessment of therapeutic effects, tumor growth and survival study, injected activity was either 4.5 or 10 MBq administrated and unlabeled M5A was added to obtain 150 μg/mL (15 μg of the total mass of antibody per animal). For the control group, unlabeled M5A (150 μg/mL, 100 μL) was administered. For the biodistribution experiments, a concentration of 150 μg/mL was obtained (15 μg of the total mass of antibody per animal). 

The lyophilized onalespib (AT13387) (Selleckchem, Houston, TX, USA) was dissolved in Dimethyl Sulfoxide (DMSO) to stock concentration 61.0471 mM and further diluted in 17.5% 2-hydroxypropyl beta-cyclodextrin (cyclodextrin, Sigma Aldrich, Darmstadt, Germany) before intraperitoneal (IP) injection. Onalespib was administered for four consecutive days. For the control group, DMSO in 17.5% cyclodextrin was administered IP.

### 2.3. In Vivo Colorectal Xenograft Models

The *in vivo* studies were performed in accordance with current Swedish laws and regulations with the approval of the Uppsala Committee of Animal Research Ethics. Studies were designed to comply with replace, reduce, and refine (3R) animal study principles while maintaining the quality of data, with the approval of the Uppsala Committee of Animal Research Ethics. Female Balb/c nude mice (age = 6–8 weeks, weight = 16–20) were housed under standard conditions and fed ad libitum. A total of 70 mice were involved in the present study. Of these, 3 were used for the pilot study to select and optimize the tumor xenograft, 35 were used in the biodistribution study and molecular assessment, and 32 were used in the therapy study. Either 5 × 10^6^ HT55 cells in PBS or 5 × 10^6^ SNU1544 cells in PBS inoculated subcutaneously into the flank. The tumor takes in both xenograft models were 100%. After tumors were established, body weight and tumor growth were monitored every other day. Tumor diameter was measured using a digimatic caliper (Mitutoyo, Sweden) and volume was calculated as (Length × Width^2^) × 0.5236. Mice with tumor sizes 60–300 mm^3^ were selected for the studies. 

### 2.4. Radiolabeled M5A Biodistribution Studies

#### 2.4.1. Xenograft Characterizations

To verify antigen selectivity after labeling in physiological conditions as well as selecting the most suitable xenograft model, a small biodistribution study with iodinated M5A (^125^I-M5A) was performed in mice bearing either HT55 or SNU1544 xenografts (*N* = 3). Eleven days after tumor cells inoculation, each animal was administered approximately 250 kBq ^125^I-M5A (unlabeled M5A was added to obtain 150 μg/mL) by an IV injection of a volume of 100 μL into the tail vein (15 μg of total antibody/mouse). Twenty-four hours post-injection, animals were euthanized by a mixture of ketamine (10 mg/mL) and xylazine (1 mg/mL) at a dose of 0.2 mL per 10 g of body weight (IP), followed by heart puncture. Blood was aspirated, and organs of interest were excised and weighed. Average tumor weight was approximately 160 mg at the study endpoint. The radioactivity (uptake) of ^125^I-M5A of blood and organs together with an injection standard of 100 μL (approximately 250 kBq) was measured in a gamma counter (1480 Wizard 3′, Wallace, Finland). The injected activity of ^125^I-M5A for each animal was calculated by subtracting the residual activity in the syringe from the standard injected dose. Radioactivity uptake in the organ was calculated as percent of injected activity per gram of tissue (%ID/g).

#### 2.4.2. ^177^Lu-DOTA-M5A Biodistribution Studies

After selecting the HT55 tumor xenograft for further investigation, two biodistribution studies were conducted with ^177^Lu-DOTA-M5A (*N* = 27). Thirteen days after HT55 tumor cell inoculation, each animal was administered approximately 400 kBq ^177^Lu-DOTA-M5A (150 μg/mL) by an IV injection of 100 μL into the tail vein (15 μg of total antibody/mouse). Animals were euthanized 3 h (*n* = 4), 24 h (*n* = 4), 48 h (*n* = 4), 96 h (*n* = 4), and 168 h (*n* = 3) post-injection, blood was aspirated and organs of interest were excised and weighed. The radioactivity (uptake) of ^177^Lu-DOTA-M5A of blood and organs together with an injection standard of 100 μL (approximately 400 kBq) were measured in a gamma counter. The injected activity of ^177^Lu-DOTA-M5A for each animal was calculated as described earlier. The serum was separated on half of each blood sample using microvette tubes (Microvette^®^ 500 Serum, Merck, Darmstadt, Germany) for further assessment of soluble CEA level.

To further assess radioconjugate uptake in tumor and vital organs in animals bearing smaller tumors (seven days after tumor cells inoculation) and receiving either ^177^Lu-DOTA-M5A (4.5 MBq), or the combination of 4.5 MBq of ^177^Lu-DOTA-M5A and onalespib, a second biodistribution study was then performed, where the treatment scheme of the therapy study was imitated (*N* = 8) but all animals were euthanized (as described earlier) two days after the regime finished (96 h post-administration of ^177^Lu-DOTA-M5A). Tumors were 137 ± 32 mm^3^ in size at the time of radioconjugate administration. Blood was aspirated, tumor, liver, kidney, and spleen samples were collected. The radioactivity (uptake) of ^177^Lu-DOTA-M5A of blood and vital organs together with an injection standard of 100 μL (approximately 4.5 MBq) were measured in a gamma counter. The injected activity of ^177^Lu-DOTA-M5A for each animal was calculated as described earlier. The serum was separated from half of each blood sample using microvette tubes for further assessment of soluble CEA level.

#### 2.4.3. SPECT/CT Imaging

Whole body scans of animals receiving either 4.5 MBq of ^177^Lu-DOTA-M5A or the combination of 4.5 MBq of ^177^Lu-DOTA-M5A and onalespib were performed 72 h post-radioconjugate injection using NanoScan SPECT/CT (Mediso Medical Imaging Systems Ltd., Budapest, Hungary) to visualize and compare the biodistribution of ^177^Lu-DOTA-M5A. Mice were anesthetized by inhalation of 2–4% isoflurane in oxygen during the scan. A computed tomography (CT) acquisition was first carried out to position the body of animals in the camera at the following parameters: Semi circle field of view (FOV), projections 480; X-ray, 50 kVp and 1000 μA; binding, 1:4; acquisition time, 5 min 38 s. Subsequently, SPECT acquisition was performed in the same position for 20 min with the following parameters: acquisition 17 to 229, 101 to 124, and 50.5 to 61.7 keV. CT raw files were reconstructed by Filter Back Projection (FBP). SPECT raw data were reconstructed by Nucline software (3.00.020.000) using the TeraTomo 3D algorithm with 3 subsets, 48 iterations, and corrected for scatter and attenuation artifacts. SPECT and CT data were fused and analyzed using PMOD v3.508 (PMOD Technologies Ltd., Zurich, Switzerland). Coronal SPECT/CT images of the scans were presented as maximum intensity projections (MIP) in RGB color scale to obtain a visual confirmation of the biodistribution results.

#### 2.4.4. Molecular Assessment and Short-Term Toxicity via Ex Vivo Immunohistochemistry

*Ex vivo* immunohistochemistry (IHC) was performed to assess molecular response. In this experiment, the treatment scheme of the therapy study was imitated (*N* = 16, 4 per treatment group), all animals were euthanized (as described earlier) two days after the regime finished, and tumor, liver, kidney, and spleen samples were collected. Tissues were fixed in 4% buffered formaldehyde (VWR, Gdańsk, Poland), paraffin-embedded, sectioned, and deparaffinized. Embedding, sectioning, and Hematoxylin and eosin (HE)/IHC-staining was performed by the Research and Development department, Clinical Pathology at the Uppsala University hospital, Sweden. Briefly, staining was performed with a Dako Autostainer 48 (Agilent, Santa Clara, CA, USA). Antigen retrieval was performed with retrieval solution low pH (Dako K8005, Agilent, Santa Clara, CA, USA) or high pH (Dako K8004, Agilent, Santa Clara, CA, USA). Sections were immunostained with antibodies targeting cleaved PARP1 (rabbit monoclonal antibody, ab32064, Abcam, Cambridge, UK) and CEA (rabbit monoclonal antibody, ab133633, Abcam, Cambridge, UK). Signals were detected using the Envision Flex kit (Dako K8010, Agilent, Santa Clara, CA, USA). Counterstaining with hematoxylin (Histolab, Gothenburg, Sweden) was done in a Tissue-Tek Prisma (Sakura, Alphen aan den Rijn, The Netherlands). Immunohistochemical sections were manually scored according to staining intensity (negative −, weak (Grade 1, +), moderate (Grade 2, ++), or strong (Grade 3, +++) for CEA. Figure S1 illustrates the reference images. For cleaved PARP1, positive cells of four random areas of 0.2 mm^2^ per section were counted using ImageJ (1.53t, Java 1.8.0_172, NIH, Bethesda, MD, USA). In short, the areas where color deconvolution, signals in threshold window of 0–100 and area of >5 μm were counted. All histological analyses were performed blinded to the treatment group of the animal. For IHC staining, stroma and necrotic areas were excluded and only tumor tissue was scored and analyzed. For toxicity assessment in vital organs via HE staining, presence of fibrosis, necrosis, and inflammation were assessed.

For toxicity analyses, three Hematoxylin and eosin-stained sections of 0.2 mm^2^ each on the liver, kidney, and spleen were randomly chosen and presence/absence of damage was determined in a blinded manner, with consideration for the presence of necrosis and fibrosis in liver, glomeruli contraction in kidney, and necrosis and congestion in the spleen.

### 2.5. Therapy Regime, Tumor Growth, and Survival

Seven days after tumor cell inoculation, mice were randomized and individually earmarked to start the therapy regime. Animals were weighed and received one of the following treatments: (1) no treatment, control (*n* = 5), (2) DOTA-M5A, control (on day 2, *n* = 3), (3) DMSO, control (on day1–4, *n* = 4), (4) ^177^Lu-DOTA-M5A (4.5 MBq on day 2, *n* = 6), (5) ^177^Lu-DOTA-M5A (10 MBq on day 2, *n* = 3), (6) onalespib (30 mg/kg on day 1–4, *n* = 5), (7) combination therapy [^177^Lu-DOTA-M5A (4.5 MBq) on day 2 and onalespib (30 mg/kg) on day 1–4, *n* = 6]. The therapy regime is summarized in Table 1. There were no significant differences in initial tumor volume between the groups, with an average size of 166 ± 49 (range 69–274) mm^3^ for all groups. The therapy regime was four consecutive days of daily IP injection of 30 mg/kg onalespib or DMSO (control). On the second day of the therapy regime, a single dose of either 4.5 or 10 MBq ^177^Lu-DOTA-M5A (15 μg of total antibody/mouse) or DOTA-M5A (15 μg of total antibody/mouse) was injected once into the corresponding groups. Tumors were measured three times/week as the therapy started until either the study endpoint of tumor size 1.2–1.3 cm^3^ or consistent weight loss of more than 10% compared to the day of treatment initiation was reached. At the study endpoint, animals were euthanized by a mixture of ketamine (10 mg/mL) and xylazine (1 mg/mL) at a dose of 0.2 mL per 10 g of body weight (IP), followed by heart puncture. Blood was aspirated and serum was separated using microvette tubes. Tumors and livers were also harvested and preserved in formalin (VWR, Darmstadt, Germany).

To evaluate possible long-term drug-induced hepatotoxicity, serum was separated from the collected blood samples to evaluate the long-term effects of therapy on the aforementioned hepatotoxicity-related enzymes at the end-point. Collected serum samples were tested for mouse aspartate aminotransferase (AST) using an enzyme-linked immunosorbent assay (ELISA) kit (Catalog number EKU11950, Biomatik, Wilmington, DE, USA) and mouse alanine transaminase (ALT) ELISA kit (Catalog number EKN43321, Biomatik, DE, USA). ELISA assays were performed according to the manufacturer’s instructions.

### 2.6. Tumor Growth Rate Analysis

Tumor growth rates were assessed based on tumor volume measurements as described earlier. Daily tumor volume data were log-transformed and used as the dependent variable in a linear mixed model allowing for varying initial tumor size and tumor growth rate per mouse. A bootstrap procedure was used to estimate 95% confidence intervals and test the statistical significance of between-group differences [48]. Log-scale model coefficients were back-transformed to represent average daily growth rates in each treatment group for presentation. See Supplementary Document S1 for details of the analysis underlying the analysis of tumor growth rate.

### 2.7. Statistical Analysis

Statistical data analysis for experiments was performed using GraphPad Prism Version 9.4.0 (GraphPad Software Inc., La Jolla, CA, USA) and R (v 4.2.0) using the lme4 [49] and lmeresampler [49] packages to estimate mixed effects models and perform bootstrapping. Statistical comparisons between two groups were performed using Student’s t-test and statistical analysis comparing three or more groups was performed using analysis of variance (one-way ANOVA) with Tukey’s multiple comparison test. Data are presented as means ± standard deviation (SD), if not otherwise stated. *p* < 0.05 was considered significant (* *p* < 0.05, ** *p* < 0.01).

## 3. Results

### 3.1. In Vivo Biodistribution Studies of Radiolabeled M5A

#### 3.1.1. HT55 Is a Suitable Xenograft Model

**Figure 1:**
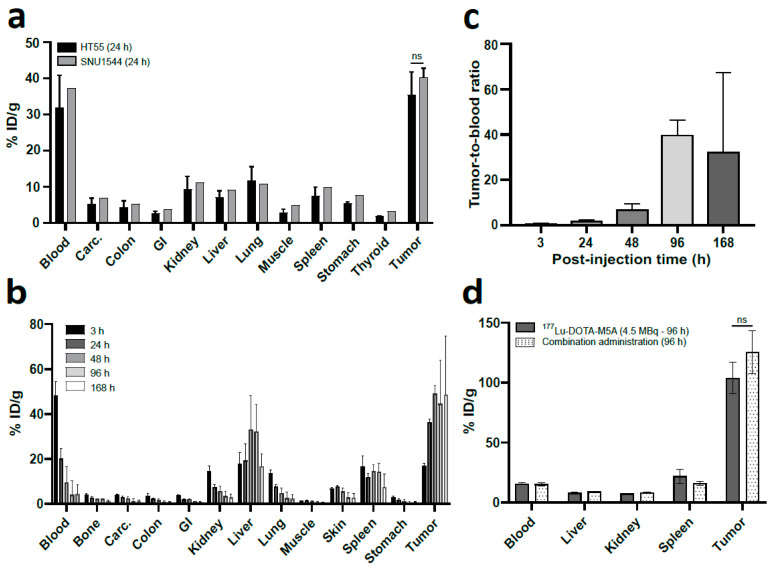
Biodistribution of ^125^I-M5A and ^177^Lu-DOTA-M5A in Balb C nude mice. (**a**) Biodistribution of ^125^I-M5A as %ID/g 24 h post-injection in Balb C nude mice bearing HT55 tumors (in black) or SNU1544 tumors (in grey), *n* ≥ 1. Thyroid uptake was presented as %ID/organ. (**b**) Biodistribution of ^177^Lu-DOTA-M5A as %ID/g in Balb C nude mice bearing HT55 tumors (409 ± 0.24 mg) at 3, 24, 48, 96, and 168 h post-injection, *n* ≥ 3. (**c**) Tumor to blood ratios in Balb C nude mice bearing large HT55 tumors at 3–168 h post-injection of ^177^Lu-DOTA-M5A, *n* ≥ 3. Error bars represent means ± standard deviation (SD). (**d**) Biodistribution of ^177^Lu-DOTA-M5A as %ID/g 96 h post-injection in Balb C nude mice bearing HT55 tumors (103 ± 0.02 mg) receiving either ^177^Lu-DOTA-M5A (4.5 MBq) or the combination of 4.5 MBq of ^177^Lu-DOTA-M5A and onalespib. Potential dose-limiting organs and tumors are presented. The grey bars illustrate uptakes in ^177^Lu-DOTA-M5A (4.5 MBq) group and the dotted pattern bars illustrate the uptakes in combination group, *n* ≥ 4.

Biodistribution of the anti-CEA ^125^I-M5A mAb was first performed to evaluate the tumor uptake in HT55 and SNU1544 xenografts (Figure 1a). ^125^I-M5A mAb tumor uptake 24 h post-injection in HT55 and SNU1544 tumor xenografts were 36 ± 6 injected dose per gram of tissue (% ID/g) and 41 ± 3%ID/g, respectively. The tumor uptake in the two xenograft models was comparable, and HT55 was chosen for continued *in vivo* assessments.

#### 3.1.2. *In Vivo* Tumor Targeting was Confirmed

An extended biodistribution study was conducted using ^177^Lu-DOTA-M5A mAb on HT55 xenografts (409 ± 0.24 mg, Figure 1b). Tumor uptake was 17 ± 1%ID/g at 3 h post-injection and peaked at 49 ± 4%ID/g at 48 h, remaining steady until 168 h. Blood activity decreased with time. Uptake in organs peaked at 3 h for the kidney and spleen, and at 48 h for the liver, decreasing at later time points. Tumor to blood ratio was 0.35 ± 0.035 at 3 h and peaked at 39.3 ± 6.9 at 96 h (Figure 1c).

#### 3.1.3. Low Uptake in Organs at Risk, SPECT/CT Imaging

**Figure 2:**
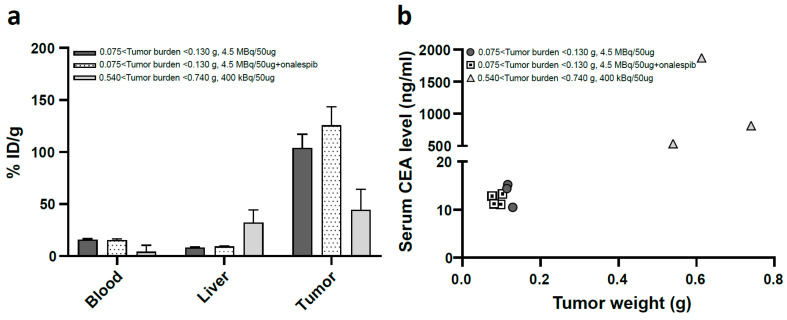
Blood CEA levels in relation to antibody tumor and liver uptakes. (**a**) Biodistribution of ^177^Lu-DOTA-M5A as %ID/g 96 h post-injection in Balb C nude mice bearing HT55 tumors (103 ± 0.02 mg) receiving ^177^Lu-DOTA-M5A (4.5 MBq, dark grey bars), the combination (dotted bars), and HT55 tumors (640 ± 103 mg, light grey bars). Blood, liver, and tumors are presented. The error bars represent the means ± SD, *n* ≥ 4. (**b**) Blood CEA levels in relation to tumor size. Dark grey circles and dotted squares represent animals bearing tumors of 103 ± 0.02 mg receiving ^177^Lu-DOTA-M5A (4.5 MBq) or the combination, *n* = 3. Triangles represent animals bearing tumors of 640 ± 103 mg receiving ^177^Lu-DOTA-M5A (400 kBq), *n* = 3.

**Figure 3:**
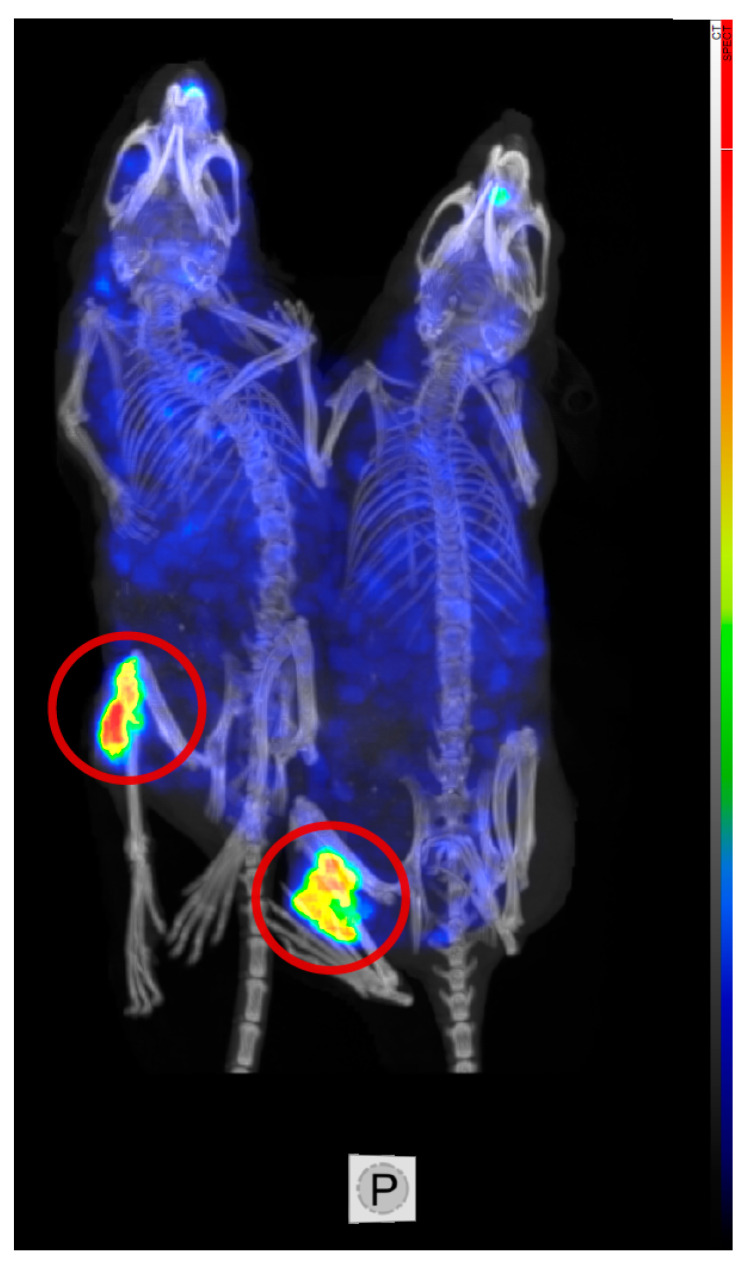
SPECT/CT image of two animals receiving ^177^Lu-DOTA-M5A (4.5 MBq), on the left, or combination of ^177^Lu-DOTA-M5A (4.5 MBq) and onalespib (on the right) 72 h post-injection of radio-conjugate. Tumors were on the left flanks and are marked with red circles. Animals had equally large tumors. The color scale shows SPECT image and grey scale shows CT image.

Since previous studies have demonstrated an association between tumor burden and amount of soluble CEA in the blood [50], which can eventually alter the biodistribution profile, a second biodistribution study was performed on mice bearing smaller tumors (137 ± 32 mm^3^, 103 ± 0.02 mg, Figure 1d), representative of the therapeutic setting. As expected, uptake in liver, kidneys, and spleen was significantly lower in this experimental setting, with subsequently higher tumor uptake. Liver, kidney, and spleen uptakes 96 h post-injection were 8.7 ± 0.7%, 8 ± 0.5%ID/g, and 18.7 ± 4.8%ID/g, respectively. The average tumor uptake was 116 ± 19%ID/g, and did not differ significantly between two groups. ELISA assessment of blood CEA levels demonstrated a lower blood CEA level (12.6 ± 1.8 ng/mL) in animals with smaller tumor burden (0.1 ± 0.02 g) compared to animals with larger tumor burden [(0.64 ± 0.1 g, 1074 ± 700 ng/mL), Figure 2]. SPECT/CT imaging of animals receiving 4.5 MBq of ^177^Lu-DOTA-M5A or combined administration of ^177^Lu-DOTA-M5A (4.5 MBq) and onalespib (72 h post-injection of radioconjugate) demonstrated high tumor uptakes and no detectable uptake in vital organs (Figure 3).

#### 3.1.4. Therapeutic Effects at Molecular Level and No Detected Short-Term Toxicity

**Figure 4:**
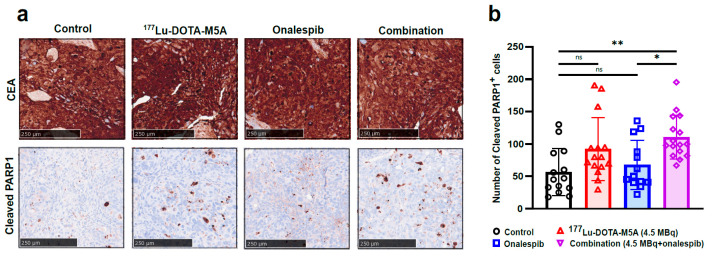
Immunohistochemical staining of tumor tissues from untreated animals and treated animals with ^177^Lu-DOTA-M5A (4.5 MBq), onalespib, and the combination thereof 48 h after treatment ends (96 h post-radioconjugate injection). (**a**) Representative images of tumor sections stained for CEA (top row) and cleaved PARP1 (down row). The scale bar is 250 μm, *n* ≥ 4. (**b**) Quantification of cleaved PARP1+ cells. Black circles are positive cell counts of untreated control group, upward red triangles represent cell counts for ^177^Lu-DOTA-M5A treatment group, blue squares represent cell counts for onalespib treatment group, and purple rhombuses represent cell counts for combination treatment group. The error bars represent the means ± SD (* *p* < 0.05, ** *p* < 0.01), *n* ≥ 4. ns = not significant.

Immunohistochemical-stained sections demonstrated a significant increase in cleaved PARP1+ cells in the combination-treated tumors compared to onalespib-treated tumors 48 h after end of treatment (96 h post-radioconjugate injection). No significant differences were observed between ^177^Lu-DOTA-M5A and combination therapy (Figure 4). CEA was strongly expressed in the tumors of all groups according to CEA expression grades (Figure 4a and Figure S1) and no CEA expression alteration attributable to the administered therapeutic regimen was detected. Hematoxylin and eosin staining of the vital organs, liver, kidney, and spleen, assessing the presence of necrosis and fibrosis in liver, glomeruli contraction in kidney, and necrosis and congestion in spleen (Figure S2) at the same time point did not indicate any toxicity.

### 3.2. In Vivo Therapy Study

#### 3.2.1. Combination Therapy Decreased Tumor Growth Rate

**Figure 5:**
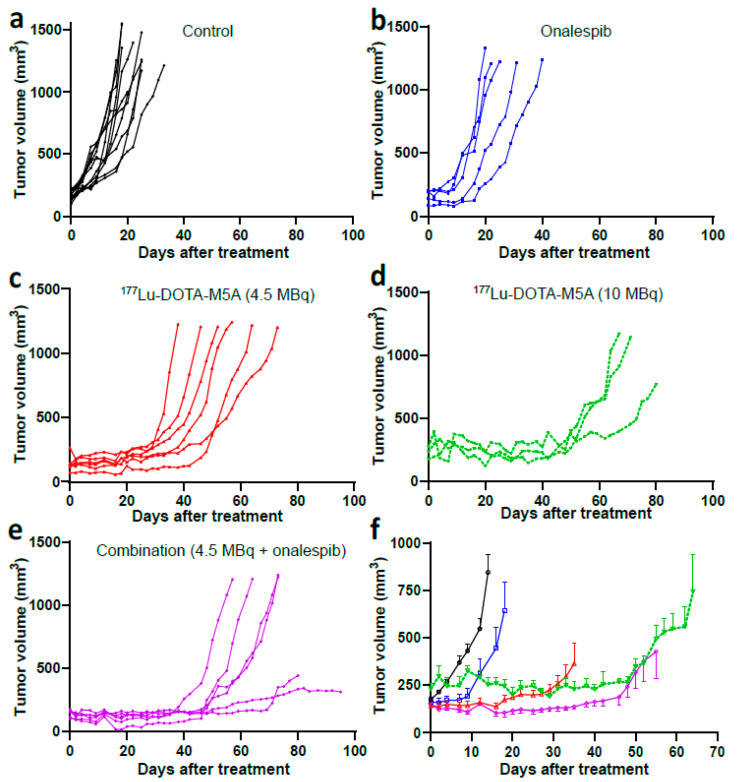
Tumor growth curves. (**a**–**e**) Individual tumor growth curves. The individual tumor growth of control (black circles/lines), onalespib (blue squares/lines), 4.5 MBq of ^177^Lu-DOTA-M5A (red upward triangles/lines), 10 MBq of ^177^Lu-DOTA-M5A (green downward triangles/dashed lines), and combination therapy (purple rhombuses/lines) are illustrated, *n* ≥ 3. (**f**) Tumor growth over time. The same color/shape codes were applied (mean of tumor size at each time, error bars represent SEM. *n* ≥ 3). The graph was cut when the first animal in the group was euthanized. Graphs display tumor volume (mm^3^) on *y* axis and days after treatment on *x* axis.

In a xenograft model with human colon carcinoma HT55, the therapeutic effects of ^177^Lu-DOTA-M5A and onalespib were investigated alone and in combination. The tumor growth rates demonstrated therapeutic benefits for ^177^Lu-DOTA-M5A and the combination therapy groups. The daily tumor growth rates are listed in Table 2, the daily tumor growth curves and a comparison summary are illustrated in Figure 5. The daily tumor growth rate did not exhibit any statistically significant differences among the control groups. As a result, they were consolidated into a singular group for further analysis. While monotherapy with onalespib reduced the tumor growth rate, the impact did not attain statistical significance compared to controls. However, monotherapy with 4.5 MBq and 10 MBq of ^177^Lu-DOTA-M5A reduced tumor growth rates by a factor of 2.4 and 3.4 times, respectively. Combination therapy of 4.5 MBq of ^177^Lu-DOTA-M5A and onalespib significantly reduced tumor growth rate compared to monotherapies and controls by a factor of 4, 1.6, and 3.5 compared to control group, ^177^Lu-DOTA-M5A (4.5 MBq), and onalespib, respectively. The tumor growth rates of combination therapy (4.5 MBq of ^177^Lu-DOTA-M5A and onalespib) and 10 MBq of ^177^Lu-DOTA-M5A were not significantly different (Figure 5d,e). No complete tumor remission was observed. Serum levels of hepatotoxicity-related enzymes were below the detection limit for all samples (0.312 ng/mL for AST and 1.56 ng/mL for ALT).

#### 3.2.2. Combination Therapy Increased the Median Survival

**Figure 6:**
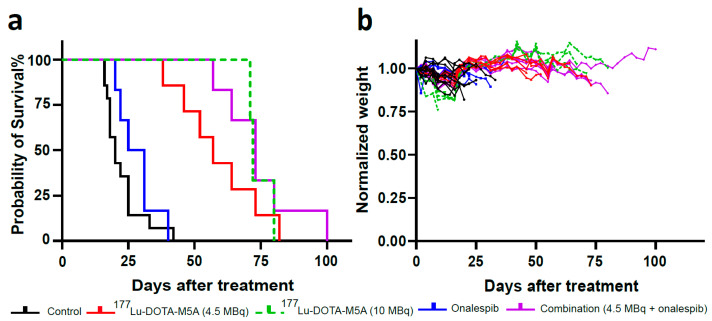
Survival curves and animal weights of the therapy study. (**a**) Survival curves. The Kaplan-Meier survival of animals of control (black curve), onalespib monotherapy (blue curve), 4.5 MBq of ^177^Lu-DOTA-M5A monotherapy (red curve), 10 MBq of ^177^Lu-DOTA-M5A monotherapy (green dashed curve), and combination therapy (purple curve) are illustrated, *n* ≥ 3. (**b**) Individual weight of animals over the therapy study. The same color code was applied, *n* ≥ 3.

Next, the *in vivo* data was further analyzed for median and maximum survival (Figure 6a and Table 2). Onalespib alone increased animal survival by 25%, while 4.5 or 10 MBq of ^177^Lu-DOTA-M5A increased median survival by 175% and 260% compared to control. Combination therapy of 4.5 MBq ^177^Lu-DOTA-M5A and onalespib improved median survival by 265% compared to control. The median survival of combination treated animals (4.5 MBq of ^177^Lu-DOTA-M5A and onalespib) and monotherapy with 10 MBq of ^177^Lu-DOTA-M5A was comparable; moreover, the maximum survival of combination-treated animals was 100 days, while it was 80 days for animals treated with 10 MBq of ^177^Lu-DOTA-M5A. Thus, onalespib potentiated the therapeutic effects of 4.5 MBq ^177^Lu-DOTA-M5A to that of more than the double dose (10 MBq) of ^177^Lu-DOTA-M5A as a monotherapy. The individual mouse weights are presented in Figure 6b.

## 4. Discussion

RIT has demonstrated significant potential for cancer therapy [51]. The anti-CEA T84.66 monoclonal antibody (mAb) is a high affinity mAb that targets CEA-positive disease, and radiolabeled variants have demonstrated specificity in clinical trials alone and when combined with chemotherapy drugs [13,14,15,52]. A humanized version, hT84.66- M5A, has been developed and a clinical trial with beta-emitter ^90^Y has shown therapeutic potential [14]. While ^177^Lu is a well-established radionuclide for cancer therapy, it has not previously been assessed with M5A for potential in RIT of solid tumors.

Despite its promise, RIT has some limitations for solid tumors due to abnormal blood flow and heterogeneous antigen expression. As a result, combination therapies have been explored to enhance therapeutic effects and minimize toxicity [53,54], where CEA targeting combined with chemotherapy or immunocytokines has shown some promise [15,18,19,22,23]. Moreover, HSP90 inhibitors have been studied in preclinical and clinical trials, either alone or with other therapies such as chemotherapy or radiation [55]. Onalespib, a second-generation HSP90 inhibitor, has demonstrated radiosensitizing properties in preclinical studies when combined with external beam radiation, ^177^Lu-DOTA-M5A (*in vitro*) [27,38], or ^177^Lu-DOTATATE in tumor xenografts [56]. The combination of CEA-targeted RIT with HSP90 inhibition using onalespib is, thus, an intriguing therapeutic strategy that warrants further investigation. In this paper, following the successful *in vitro* characterization of ^177^Lu-DOTA-M5A [27], we have assessed the therapeutic strategy of ^177^Lu-DOTA-M5A in combination with the HSP90 inhibitor onalespib *in vivo* for the first time.

Initial evaluation of tumor uptake using ^125^I-M5A in human colon adenocarcinoma SNU1544 and human colon carcinoma HT55 xenografts demonstrated that both models had high and comparable tumor uptake values above 35%ID/g (Figure 1). An extended biodistribution study was then performed using ^177^Lu-DOTA-M5A in HT55 tumor-bearing mice, illustrating high and stable tumor uptake over seven days, in line with previous studies [25,57]. The HT55 tumor xenograft model was selected for subsequent therapeutic investigations of ^177^Lu-DOTA-M5A and/or onalespib treatments.

In the context of a biodistribution study using ^177^Lu-DOTA-M5A, the liver exhibited the highest percentage injected dose per gram of tissue (%ID/g) compared to other organs, which is consistent with the fact that the liver’s reticuloendothelial system is responsible for clearing circulating CEA and IgGs [58]. The liver’s high internalization and catabolism is further demonstrated by the comparison of liver uptake between ^125^I- and ^177^Lu-labeled antibodies, where the latter had much higher uptake values than iodine (Figure S3). It is likely that the higher levels of soluble circulating CEA in animals with larger tumor burdens (Figure 2) may have led to more formation of ^177^Lu-DOTA-M5A:CEA complexes, resulting in higher uptake in the liver (approximately 32%ID/g at 96 h post-injection) than in animals with smaller tumors. This theory is supported by the lower liver uptake observed in mice with smaller tumors, and consequently lower and more clinically relevant CEA blood levels [59,60], resulting in lower than 10%ID/g liver uptake (as seen in Figure 1b,d). As smaller tumors were used in the subsequent therapy study, these results suggest a low risk of ^177^Lu-DOTA-M5A induced hepatotoxicity in the therapy study.

In the therapy study, the effects of ^177^Lu-DOTA-M5A and HSP90 inhibitor onalespib, alone or in combination, were evaluated (Figure 5 and Figure 6 and Table 2). The results showed that monotherapies reduced tumor growth rate and increased median and maximum survival compared to controls, with ^177^Lu-DOTA-M5A (in both doses) being more effective than onalespib in the present settings. Moreover, tumor growth rate was significantly decreased in the combination therapy compared to the corresponding monotherapies, further reducing tumor growth rates by 1.6 times compared to monotherapy of 4.5 MBq of ^177^Lu-DOTA-M5A group and 3.5 times compared to HSP90 inhibitor onalespib group. Animals treated with 10 MBq of ^177^Lu-DOTA-M5A exhibited comparable suppression of tumor growth to those receiving a combination therapy with a significantly lower dose (4.5MBq) of ^177^Lu-DOTA-M5A. It should be noted that this analysis assumes constant growth rates, which appeared to be the case in control and onalespib mice. In mice treated with ^177^Lu-DOTA-M5A, however, tumor growth tended to be suppressed in early measurements, while accelerating further along in the experiment (see Figure 5). Immunohistochemical analysis of harvested tumors showed significant expression of CEA (in all groups) and increased apoptosis and karyorrhexis in ^177^Lu-DOTA-M5A or the combination treatment groups. These findings support previous studies showing that radiation-induced DNA damage causes apoptosis and karyorrhexis [61].

As for survival data for monotherapies, onalespib alone slightly increased median survival (25 days) compared to control (20 days), while 4.5 or 10 MBq of ^177^Lu-DOTA-M5A substantially increased median survival by 175% (55 days) and 260% (72 days), respectively (Figure 6). These beneficial radioimmunotherapy results are in line with the initial phase I clinical trials using ^90^Y-labeled chimeric antibody T84.66 [24,62] and the recent phase I clinical trial using the same radionuclide for labeling a humanized version of the antibody (^90^Y-DOTA-M5A) for advanced CEA producing cancers [14]. Although RIT with antibodies against CEA shows promise, the therapeutic effects can be diminished by varying levels of CEA expression in CRCs and heterogeneity in tumor bulk [63,64]. Hence, a more potent therapy approach is needed to overcome these limitations and a suitable combination therapy could be transformative.

Even though monotherapies were effective, the real promise was shown in the combination treatments. In the present study, combination therapy demonstrated a 265% increased median survival (73 days) compared to control, corresponding to a 192% improvement in survival compared to onalespib monotherapy and 33% compared to 4.5 MBq of ^177^Lu-DOTA-M5A monotherapy. Molecular assessment demonstrated that the number of apoptotic cells was greater in tumors treated with ^177^Lu-DOTA-M5A either as a standalone therapy or in combination, significantly higher for the combination group compared to the onalespib monotherapy and control groups. Moreover, the combination therapy was so effective that retained therapeutic effects could be achieved with only half the dose of administered ^177^Lu-DOTA-M5A, potentially decreasing toxicity and improving therapeutic efficiency. These findings support previous studies combining ^90^Y-cT84.66 with chemotherapy agents in CEA-expressing tumors [19,22,23] and suggest that ^177^Lu-DOTA-M5A and onalespib may be a promising combination therapy for CRCs.

Using full-sized antibodies like M5A in RIT can increase tumor uptake [65], but it also increases the risk of high radiation dose to normal organs including bone marrow and liver, as liver processes antigen-antibody complexes [66]. Moreover, while onalespib is a second-generation HSP90 inhibitor with relatively low hepatotoxicity, hepatotoxicity as a dose-limiting factor must nonetheless be considered in combination therapies [67]. In the present study, AST and ALT enzyme levels were, therefore, closely investigated, demonstrating that the values of both hepatotoxic-related enzymes in blood were below the detection limit of the ELISA assays. The study found no hepatotoxicity or significant weight loss in animals receiving monotherapies and the combination therapy thereof, indicating the potential benefit of this combination therapy for future therapies.

## 5. Conclusions

The ^177^Lu-DOTA-M5A is a promising novel radioconjugate with potential for RIT of CEA-expressing CRCs. In addition, onalespib can further potentiate the therapeutic effects of ^177^Lu-DOTA-M5A, prolong the survival, further suppress the tumor growth and enhance tumor cells apoptosis. Henceforth, using ^177^Lu-DOTA-M5A with HSP90 inhibitors as combination therapies may be a feasible therapy approach for metastatic colorectal cancers to overcome the antitumor therapy resistance, and may have the potential to widen the targetable patient population.

## Figures and Tables

**Table 1:** Summary of therapy regime. X represents either IP or IV injections. Hyphen represents no intervention.

Groups\Days	−6	1	2	3	4	Termination of First/Last Mouse (Days after Treatment Start)
**Control (no treatment)**	TI *	-	-	-	-	16/25
**Control (DOTA-M5A)**	TI	-	X	-	-	20/33
**Control (DMSO)**	TI	X	X	X	X	18/22
**^177^Lu-DOTA-M5A (4.5 MBq)**	TI	-	X	-	-	38/82
**^177^Lu-DOTA-M5A (10 MBq)**	TI	-	X	-	-	67/80
**Onalespib**	TI	X	X	X	X	20/40
**Combination (4.5 MBq of ^177^Lu-DOTA-M5A and onalespib)**	TI	X	X	X	X	57/100

* TI represent tumor inoculation day.

**Table 2:** Summary of *in vivo* tumor growth rates and survival.

Treatment	Median Survival (days)	Maximum Survival (days)	Average Daily Tumor Growth Rate (%, 95% CI)
**Control (no treatment)**	20	33	8.95 (7.67–10.14)
**^177^Lu-DOTA-M5A (4.5 MBq)**	55	73	3.63 (1.35–5.89)
**^177^Lu-DOTA-M5A (10 MBq)**	72	>80 *	2.6 (0.24–4.82)
**Onalespib**	25	40	7.99 (4.95–10.87)
**Combination (4.5 MBq of ^177^Lu-DOTA-M5A and onalespib)**	73	>100 **	2.25 (−0.17–4.60)

* The last animal in ^177^Lu-DOTA-M5A group was euthanized according to the ethical permission with the tumor size of 770 mm^3^; ** The last two animals in the combination therapy group were euthanized according to the ethical permission with the tumor size of 307 and 691 mm^3^.

## Data Availability

The datasets generated for this study are available on request to the corresponding author.

## Supplementary Materials

The following supporting information can be downloaded at: https://www.mdpi.com/article/10.3390/cancers15174239/s1, Figure S1: IHC grading reference. This figure displays manually graded immunohistochemical staining sections targeting CEA. The scale bar is 50 μm; Figure S2: Hematoxylin and eosin staining of vital organs. This figure displays representative images of liver, kidney and spleen of untreated animals (control), animals treated with ^177^Lu-DOTA-M5A (4.5 MBq), onalespib or combination of ^177^Lu-DOTA-M5A (4.5 MBq) and onalespib, *n* = 3 per treatment group.; Figure S3: Biodistribution of ^177^Lu-DOTA-M5A and ^125^I-M5A as %ID/g in Balb C nude mice bearing HT55 xenografts 24 h post-injection as %ID/g. Error bars represent the means ± SD), *n* ≥ 2; Supplementary Document S1: Tumor growth analysis notebook. This document provides an accounting of the analysis steps relating to tumor growth rates in the paper “Enhanced therapeutic effects of ^177^Lu-DOTA-M5A in combination with heat shock protein 90 inhibitor onalespib in colorectal cancer xenografts”.
