# Supplementary material for: Enhanced Therapeutic Effects of 177Lu-DOTA-M5A in Combination with Heat Shock Protein 90 Inhibitor Onalespib in Colorectal Cancer Xenografts
Source: Cancers (Basel). 2023 Aug 24;15(17):4239. doi: 10.3390/cancers15174239 (PMC10486833; doi:10.3390/cancers15174239)
Supplement: Supplementary file 1 [file cancers-15-04239-s001.zip › Supplementary Decument S1.pdf]

Supplementary Document S1 - Tumor growth analysis notebook

This document provides an accounting of the analysis steps relating to tumor growth rates in the paper "Enhanced therapeutic effects of 177Lu-DOTA-M5A in combination with heat shock protein 90 inhibitor onalespib in colorectal cancer xenografts".

```
library(readxl)
library(tidyverse)
library(lme4)
library(lmeresampler)

options(scipen = 999,
        digits = 5)

set.seed(42)

# Change this to the file location
file_path <- "221028-pooled data_clean.xlsx"

d0 <- read_excel(file_path,sheet = "Cleaner data")

d <- d0 |>
  mutate(across(.cols = -c(group,type,study,euth_reason,animal),
                .fns = function(x){
                  as.numeric(gsub("\\*","",x))
                })) |>
  pivot_longer(-c(group,type,study,excluded,euth_reason,animal),names_to = "day") |>
  filter(!is.na(value),
         excluded == 0) |>
  mutate(day = as.numeric(day),
         log_value = log(value),
         id = factor(paste(group,animal)))
```

A quick look at the data (straight volumes and log transformed)

```
d |>
  ggplot(aes(x=day,y=value,group=id)) +
  geom_line() +
  facet_wrap(~group)
```

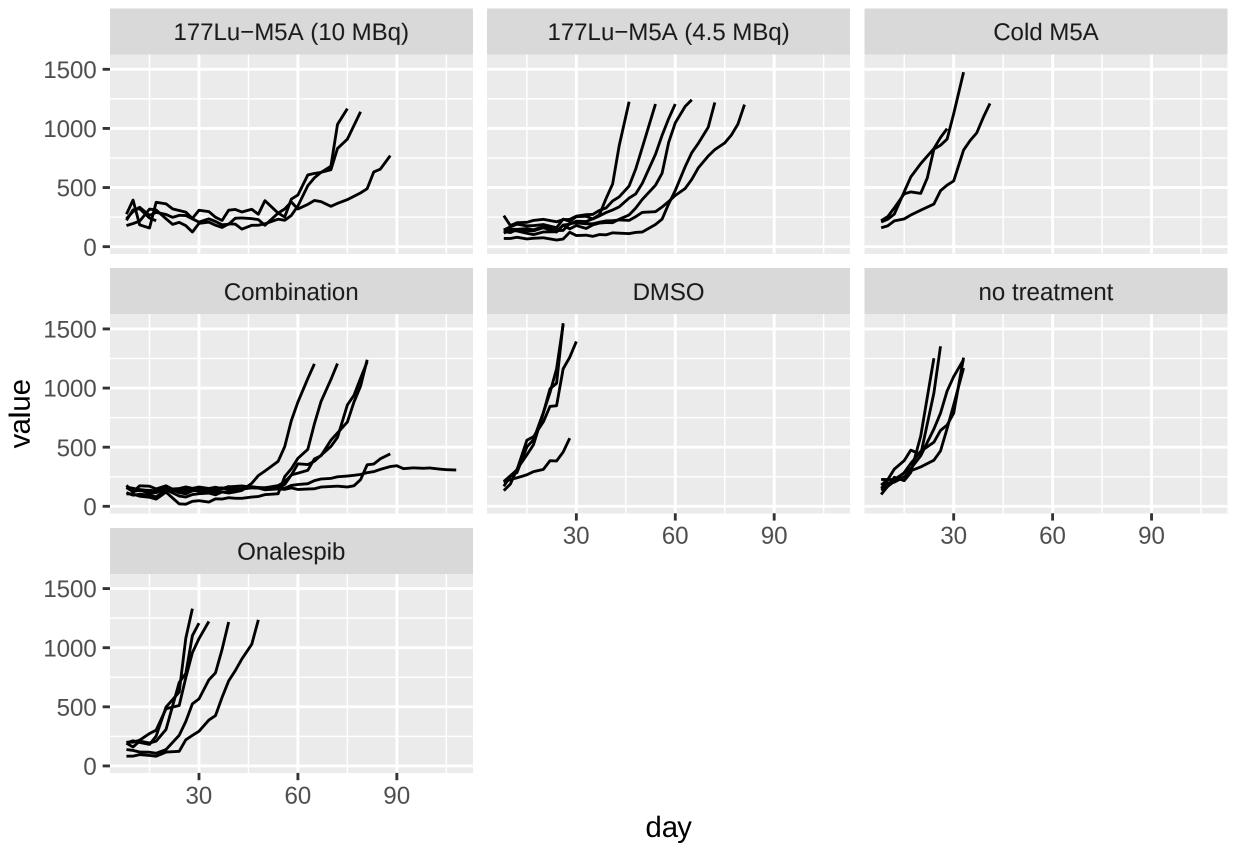


```r
d |>
  ggplot(aes(x=day,y=log_value,group=id)) +
  geom_line() +
  facet_wrap(~group)
```

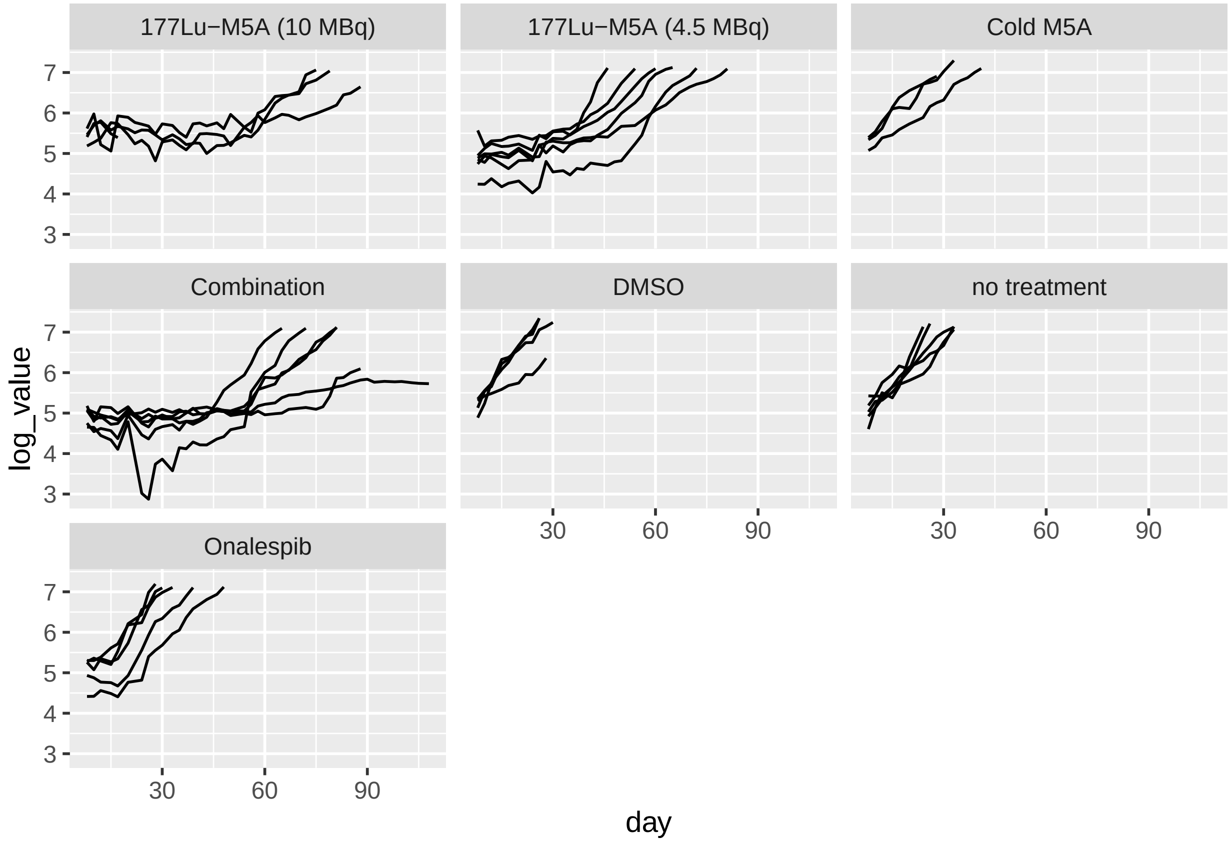

First question: Are controls similar enough to be pooled in the analysis?

```
ctrl_data <- filter(d,type == "control")

ctrl_data |>
  ggplot(aes(x=day,y=value,color=group,group=id)) +
  geom_line()
```

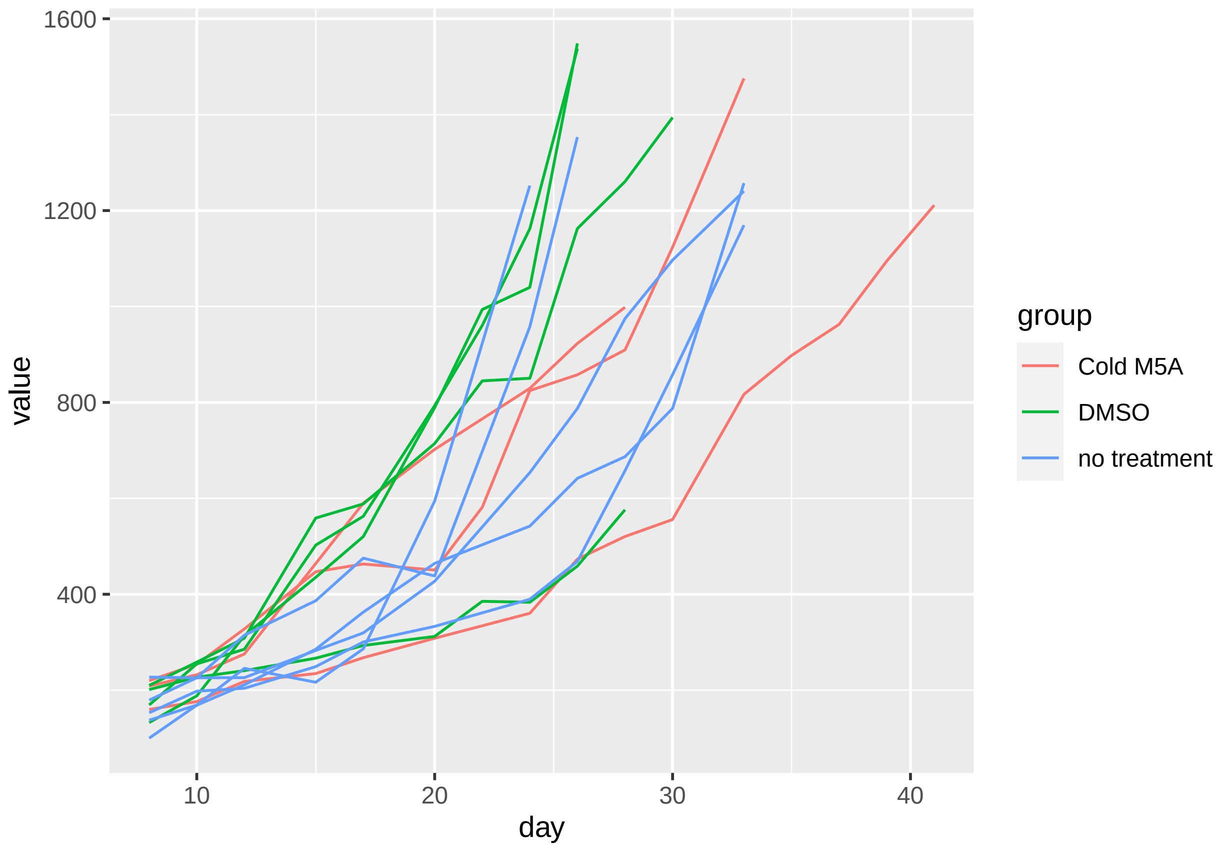


```
ctrl_data |>
  ggplot(aes(x=day,y=log_value,color=group,group=id)) +
  geom_line()
```

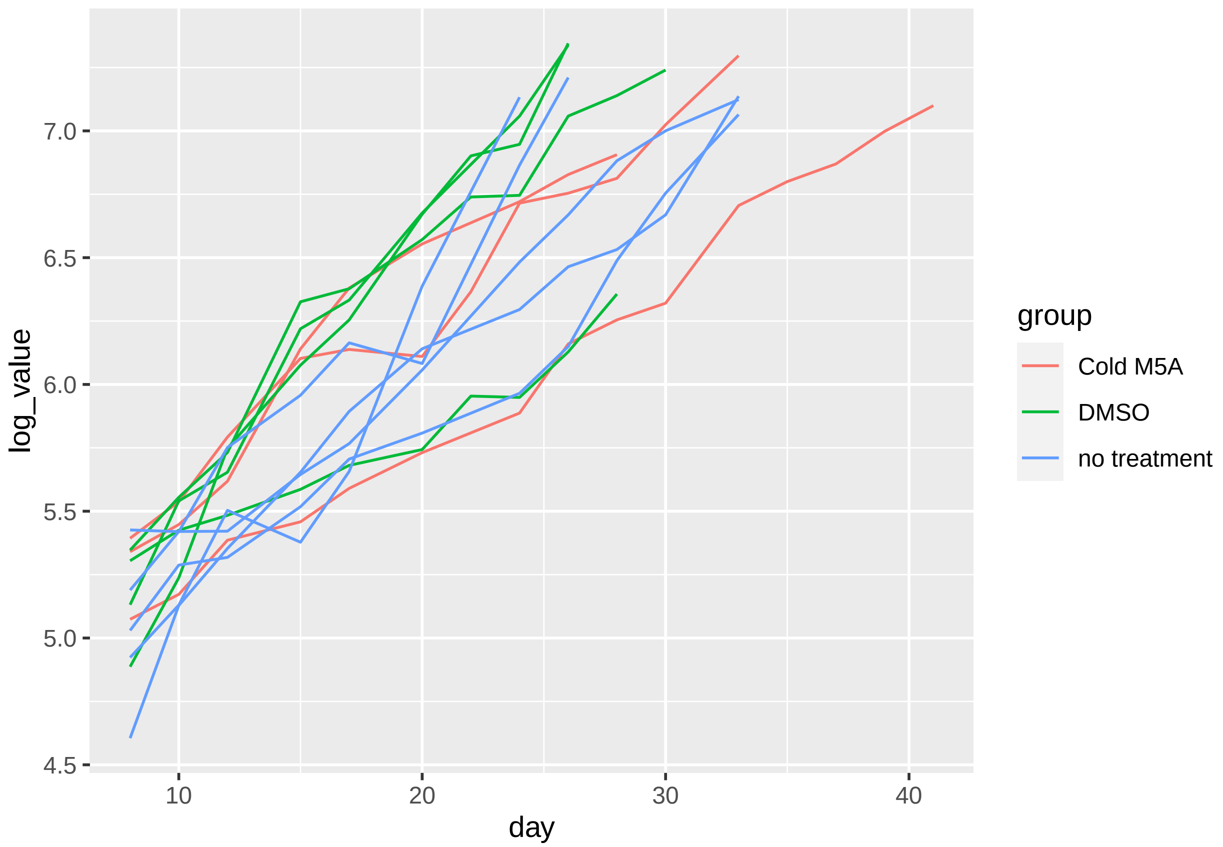


```
# Linear fit on Log transformed data
ctrl_mod1 <- lmer(log_value ~ day + day:group + (1 + day | id),
                  data = ctrl_data)
```

```
summary(ctrl_mod1)
```

```
## Linear mixed model fit by REML ['lmerMod']
## Formula: log_value ~ day + day:group + (1 + day | id)
##    Data: ctrl_data
##
## REML criterion at convergence: -56.7
##
## Scaled residuals:
##     Min      1Q  Median      3Q     Max
## -2.7080 -0.5205  0.0092  0.5154  2.3514
##
## Random effects:
##  Groups   Name        Variance Std.Dev. Corr
##  id       (Intercept) 0.098114 0.3132
##           day         0.000656 0.0256   -0.83
##  Residual             0.017029 0.1305
## Number of obs: 123, groups:  id, 12
##
## Fixed effects:
##                         Estimate Std. Error t value
## (Intercept)              4.47478    0.09667   46.29
## day                      0.08816    0.01054    8.36
```

```
## day:groupDMSO          0.00859    0.01115    0.77
## day:groupno treatment -0.00694    0.01067   -0.65
##
## Correlation of Fixed Effects:
##             (Intr) day    d:DMSO
## day         -0.600
## day:grpDMSO  0.000 -0.605
## dy:grpntrtm -0.001 -0.631  0.597
```

```
# Don't find  compelling evidence that controls differ in terms of growth rate...
# So lets pool controls for comparison w treatments and set controls as the reference category

d <- d |>
  mutate(group_pool = relevel(factor(ifelse(type == "control",type,group)),
                              ref="control"))

mod1 <- lmer(log_value ~ day + day:group_pool + (1 + day | id),
             data = d)
```

```
#Check residuals of the final model

qqnorm(resid(mod1)); qqline(resid(mod1))
```

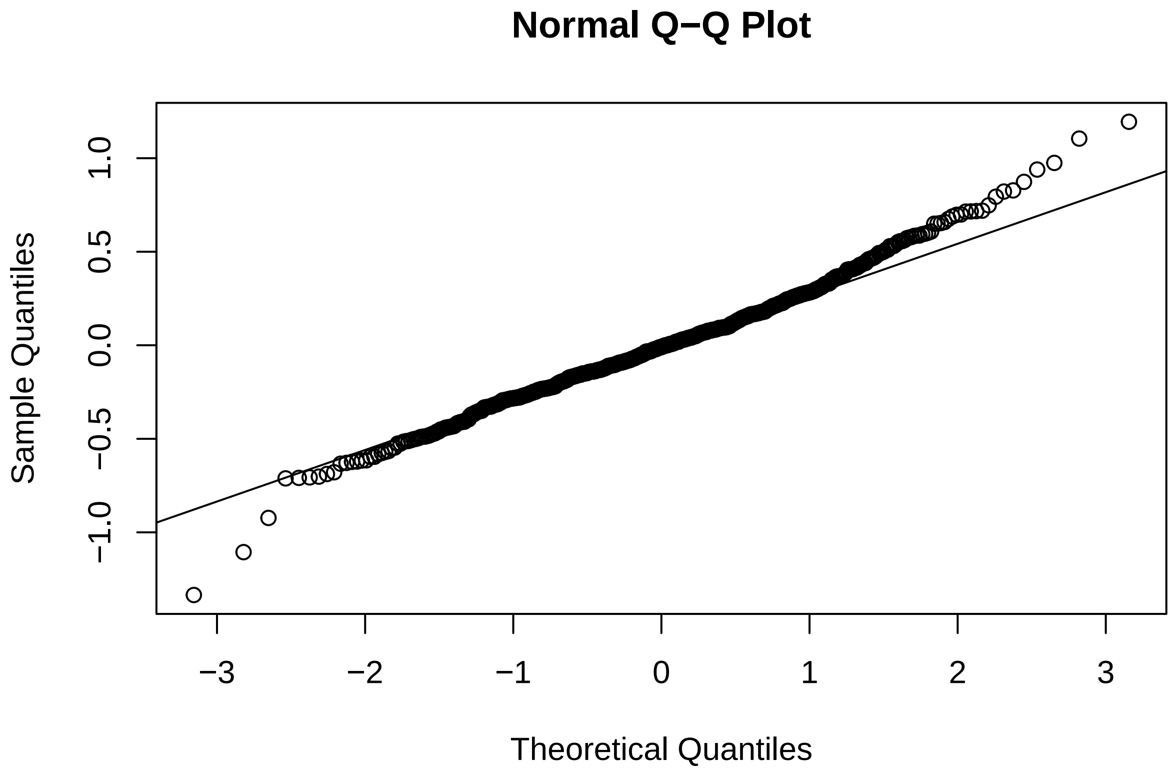


```
# Not super happy with these residuals... Might be an issue since
# some mice survive to the end of the study without getting terminated for tumor size?
# Means that we shouldn't rely on asymptotic methods for estimating CIs and such at least.
```

```r
summary(mod1)
```

```
## Linear mixed model fit by REML ['lmerMod']
## Formula: log_value ~ day + day:group_pool + (1 + day | id)
##    Data: d
##
## REML criterion at convergence: 585.6
##
## Scaled residuals:
##     Min      1Q  Median      3Q     Max
## -4.048 -0.590 -0.028  0.538  3.623
##
## Random effects:
##  Groups   Name        Variance Std.Dev. Corr
##  id       (Intercept) 0.265651 0.5154
##           day         0.000125 0.0112   -0.63
##  Residual             0.108719 0.3297
## Number of obs: 626, groups:  id, 33
##
## Fixed effects:
##                                   Estimate Std. Error t value
## (Intercept)                        4.39392    0.09747   45.08
## day                                0.08571    0.00430   19.93
## day:group_pool177Lu-M5A (10 MBq)  -0.06003    0.00626   -9.58
## day:group_pool177Lu-M5A (4.5 MBq) -0.05008    0.00531   -9.44
## day:group_poolCombination         -0.06350    0.00524  -12.12
## day:group_poolOnalespib           -0.00884    0.00633   -1.40
##
## Correlation of Fixed Effects:
##                (Intr) day    d:_177L-M5A(1M d:_177L-M5A(4M dy:g_C
## day            -0.485
## d:_177L-M5A(1M  0.098 -0.573
## d:_177L-M5A(4M  0.123 -0.680  0.438
## dy:grp_plCm     0.137 -0.694  0.445          0.526
## dy:grp_plOn     0.042 -0.540  0.361          0.427          0.433
```

```r
# All treatments except for Onalespib appear to have a robust negative effect on daily growth rates
# (Recall the critical T value here for p = 0.05 is |1.96|)
# eg exp(0.098052) = 10.3% daily growth in control group, exp(-0.078722)

# Relevel and test for differences between combination therapy and other interventions as well

d <- d |>
  mutate(group_pool_comb = relevel(factor(ifelse(type == "control",type,group)),
                              ref="Combination"))

mod1_ref_comb <-lmer(log_value ~ day + day:group_pool_comb + (1 + day | id),
              data = d)
```

```
## Warning in checkConv(attr(opt, "derivs"), opt$par, ctrl = control$checkConv, :
## Model failed to converge with max|grad| = 0.00269831 (tol = 0.002, component 1)
```

```r
summary(mod1_ref_comb)
```

```
## Linear mixed model fit by REML ['lmerMod']
```

```
## Formula: log_value ~ day + day:group_pool_comb + (1 + day | id)
##    Data: d
##
## REML criterion at convergence: 585.6
##
## Scaled residuals:
##    Min     1Q Median     3Q    Max
## -4.048 -0.590 -0.028  0.538  3.623
##
## Random effects:
##  Groups   Name        Variance Std.Dev. Corr
##  id       (Intercept) 0.265631 0.5154
##           day         0.000125 0.0112   -0.63
##  Residual             0.108719 0.3297
## Number of obs: 626, groups:  id, 33
##
## Fixed effects:
##                                        Estimate Std. Error t value
## (Intercept)                             4.39392    0.09747   45.08
## day                                     0.02222    0.00383    5.80
## day:group_pool_comb177Lu-M5A (10 MBq)   0.00347    0.00612    0.57
## day:group_pool_comb177Lu-M5A (4.5 MBq)  0.01341    0.00513    2.61
## day:group_pool_combcontrol              0.06350    0.00524   12.12
## day:group_pool_combOnalespib            0.05465    0.00623    8.77
##
## Correlation of Fixed Effects:
##                 (Intr) day    d:__177L-M5A(1M d:__177L-M5A(4M dy:g__
## day             -0.357
## d:__177L-M5A(1M -0.017 -0.539
## d:__177L-M5A(4M -0.013 -0.646  0.407
## dy:grp_pl_c     -0.137 -0.588  0.401           0.477
## dy:grp_pl_O     -0.073 -0.510  0.336           0.401           0.402
## optimizer (nloptwrap) convergence code: 0 (OK)
## Model failed to converge with max|grad| = 0.00269831 (tol = 0.002, component 1)
```

```
# All treatments except the 10 MBq have significantly higher growth than the combo treatment

# Calculate daily growth rate point estimates

cbind("log values" =fixef(mod1)[-1],
      "absolute growth rates" = c(exp(fixef(mod1))["day"],exp(fixef(mod1)["day"]+fixef(mod1))[-(1:2)]))
```

```
##                                     log values absolute growth rates
## day                                  0.0857150                1.0895
## day:group_pool177Lu-M5A (10 MBq)    -0.0600325                1.0260
## day:group_pool177Lu-M5A (4.5 MBq)   -0.0500850                1.0363
## day:group_poolCombination           -0.0634995                1.0225
## day:group_poolOnalespib             -0.0088449                1.0799
```

```
# Here we the first column is the raw model fixed effects (in log terms), second is expatiated values (

# Since we cant really rely on asymptotic methods to give us good CIs and significance tests, we'll use


mod1_boot <- bootstrap(mod1,
```

```r
                        .f = fixef,
                        hccme = "hc3",
                        aux.dist = "gamma",
                        type = "wild",
                        B = 1000)

mod1_ref_comb_boot <- bootstrap(mod1_ref_comb,
                                 .f = fixef,
                                 hccme = "hc3",
                                 aux.dist = "gamma",
                                 type = "wild",
                                 B = 1000)


#CIs vs Control as base treatment
confint(mod1_boot,type="perc")
```

```
## # A tibble: 6 x 6
##   term                               estimate   lower    upper type  level
##   <chr>                                 <dbl>   <dbl>    <dbl> <chr> <dbl>
## 1 (Intercept)                         4.39     4.22    4.57    perc   0.95
## 2 day                                 0.0857   0.0740  0.0965  perc   0.95
## 3 day:group_pool177Lu-M5A (10 MBq)   -0.0600  -0.0715 -0.0495  perc   0.95
## 4 day:group_pool177Lu-M5A (4.5 MBq) -0.0501  -0.0605 -0.0393  perc   0.95
## 5 day:group_poolCombination          -0.0635  -0.0742 -0.0516  perc   0.95
## 6 day:group_poolOnalespib            -0.00884 -0.0257  0.00666 perc   0.95
```

```r
#CIs vs Combintion as base treatment
confint(mod1_ref_comb_boot,type="perc")
```

```
## # A tibble: 6 x 6
##   term                                    estimate    lower  upper type  level
##   <chr>                                      <dbl>    <dbl>  <dbl> <chr> <dbl>
## 1 (Intercept)                              4.39     4.20    4.58   perc   0.95
## 2 day                                      0.0222   0.0159  0.0288 perc   0.95
## 3 day:group_pool_comb177Lu-M5A (10 MBq)    0.00347 -0.00450 0.0104 perc   0.95
## 4 day:group_pool_comb177Lu-M5A (4.5 MBq)   0.0134   0.00641 0.0205 perc   0.95
## 5 day:group_pool_combcontrol               0.0635   0.0532  0.0755 perc   0.95
## 6 day:group_pool_combOnalespib             0.0547   0.0419  0.0672 perc   0.95
```

```r
## generate lo/hi CIs for absolute daily growth rate per treatment group as above

b_cis <- confint(mod1_boot,type="perc")

cbind("est" = c(exp(b_cis$estimate)[2],exp(b_cis$estimate[2]+b_cis$estimate)[-(1:2)]),
      "lo" = c(exp(b_cis$lower)[2],exp(b_cis$lower[2]+b_cis$lower)[-(1:2)]),
      "hi" = c(exp(b_cis$upper)[2],exp(b_cis$upper[2]+b_cis$upper)[-(1:2)]))
```

```
##                                      est      lo     hi
## day                               1.0895 1.07679 1.1014
## day:group_pool177Lu-M5A (10 MBq)  1.0260 1.00244 1.0482
## day:group_pool177Lu-M5A (4.5 MBq) 1.0363 1.01353 1.0589
## day:group_poolCombination         1.0225 0.99983 1.0460
## day:group_poolOnalespib           1.0799 1.04952 1.1087
```
