# Supplementary figures and images for: Enhanced Therapeutic Effects of 177Lu-DOTA-M5A in Combination with Heat Shock Protein 90 Inhibitor Onalespib in Colorectal Cancer Xenografts
Source: Cancers (Basel). 2023 Aug 24;15(17):4239. doi: 10.3390/cancers15174239 (PMC10486833; doi:10.3390/cancers15174239)

Grade 0 (Negative)

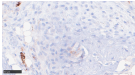

Grade 1(+)

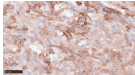

Grade 2(++)

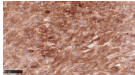

Grade 3(+++)

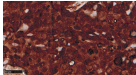

Supplement: Supplementary file 1 [file cancers-15-04239-s001.zip › Figure S1.pdf]

**Control**

**$^{177}\text{Lu}$ -DOTA-MSA**

**Onalespib**

**Combination**

**Liver**

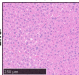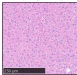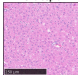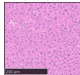

**Kidney**

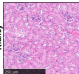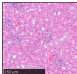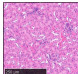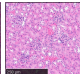

**Spleen**

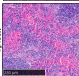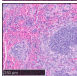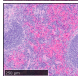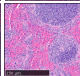

Supplement: Supplementary file 1 [file cancers-15-04239-s001.zip › Figure S2.pdf]

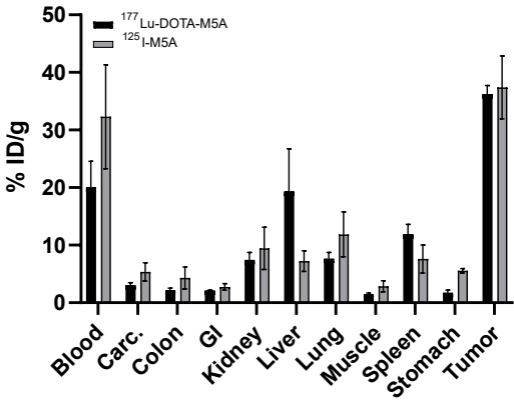

Supplement: Supplementary file 1 [file cancers-15-04239-s001.zip › Figure S3.pdf]
